# Urban rendezvous along the seashore: Ports as Darwinian field labs for studying marine evolution in the Anthropocene

**DOI:** 10.1111/eva.13443

**Published:** 2022-07-19

**Authors:** Fanny Touchard, Alexis Simon, Nicolas Bierne, Frédérique Viard

**Affiliations:** ^1^ ISEM, EPHE, IRD Université Montpellier Montpellier France; ^2^ Center of Population Biology and Department of Evolution and Ecology University of California Davis Davis California USA

**Keywords:** admixture, biological portuarization, harbors, human‐induced evolution, invasive species, marinas, ocean sprawl

## Abstract

Humans have built ports on all the coasts of the world, allowing people to travel, exploit the sea, and develop trade. The proliferation of these artificial habitats and the associated maritime traffic is not predicted to fade in the coming decades. Ports share common characteristics: Species find themselves in novel singular environments, with particular abiotic properties—e.g., pollutants, shading, protection from wave action—within novel communities in a melting pot of invasive and native taxa. Here, we discuss how this drives evolution, including setting up of new connectivity hubs and gateways, adaptive responses to exposure to new chemicals or new biotic communities, and hybridization between lineages that would have never come into contact naturally. There are still important knowledge gaps, however, such as the lack of experimental tests to distinguish adaptation from acclimation processes, the lack of studies to understand the putative threats of port lineages to natural populations or to better understand the outcomes and fitness effects of anthropogenic hybridization. We thus call for further research examining “biological portuarization,” defined as the repeated evolution of marine species in port ecosystems under human‐altered selective pressures. Furthermore, we argue that ports act as giant mesocosms often isolated from the open sea by seawalls and locks and so provide replicated life‐size evolutionary experiments essential to support predictive evolutionary sciences.

## 1 INTRODUCTION

Human‐induced environmental changes overlay natural environmental clines and can induce irreversible changes to habitats and ecosystems, either locally (e.g., through the destruction of natural habitats; Gonçalves‐Souza et al., 2020) or on a global scale (e.g., by crossing natural biogeographic barriers through human‐driven transport of species; Capinha et al., 2015). The impacts of urban areas on land have been well studied and have paved the way for the development of a fertile scientific field, named “urban science,” mostly targeting cities (e.g., Miles et al., 2021; Szulkin et al., 2020). In terrestrial urban evolution, global efforts are underway to understand adaptive responses to human‐altered environments by leveraging the repeated experiments offered by cities (Santangelo et al., 2020, 2022). However, little is known about the evolutionary effects of urbanization in coastal marine ecosystems (Alter et al., 2021).

While discussion of marine urban sciences is not new (see Bulleri, 2006), research on marine urban sciences is still in its infancy (Todd et al., 2019). It is well‐established that the impacts of human activities are numerous at sea (Halpern et al., 2019; Jouffray et al., 2020), notably in relation to habitat alterations due to sewage, aquaculture, coastal hardening, shipping activities, or wind farms. Bugnot et al. (2021) estimated that marine built constructions had direct (e.g., destruction of natural habitats) and indirect (e.g., noise or light pollution) impacts on 1.5% of the world's exclusive economic zones, a number that they found comparable to the global extent of urbanized land. The fast and global expansion of human‐made structures in the marine environment (i.e., the Ocean Sprawl, as originally coined by Duarte et al., 2013) has substantial consequences on marine ecosystems (for reviews see Bishop et al., 2017; Firth et al., 2016; Todd et al., 2019). Habitat alterations resulting from marine constructions act as urban stressors that modify the shape, structure, and substrate of the habitat, and exert specific abiotic selective pressures on the resident species (Airoldi et al., 2021; Alter et al., 2021; Bulleri & Chapman, 2010; Mineur et al., 2012). Consequently, marine urbanization has substantial evolutionary consequences, as recently reviewed by Alter et al. (2021). Despite considerable progress in recent years, more research is needed to understand the role of harbors in shaping contemporary evolution.

The impacts of marine urbanization are most pronounced in coastal areas where artificial constructions are numerous, mostly replacing natural shorelines and fragmenting natural habitats (Aguilera et al., 2020). This is well‐illustrated by a large number of commercial harbors and marinas (hereafter collectively referred to as “ports”) present in the coastal regions of many countries. For example, the French metropolitan coastline has 473 maritime ports, hosting 186,000 berths (Mission Plaisance, 2015). Ports are characterized by specific abiotic properties rarely found in natural habitats, or in other types of artificial habitats, and display particular species assemblages (Bulleri & Chapman, 2010; Connell, 2000). They are singular habitats (Box 1), with particular biotic and abiotic environment that resident species (i.e., native and nonindigenous) must cope with, resulting in evolutionary unique responses.

BOX 1 **Ports are a singular habitat for marine species**

Ports are not uniform as they vary, for instance, in time since construction, size, level of containment (e.g., some being opened to the sea and others enclosed by gates), and by the types of moored vessels and associated activities (e.g., fishing vs. leisure boats). However, they share common properties (Dafforn, 2017; Firth et al., 2016; Johnston et al., 2017; Todd et al., 2019), which are not found in natural habitats or other marine built infrastructures such as wind farms (e.g., little or no wave protection) or aquaculture sites (e.g., one dominant cultivated species).

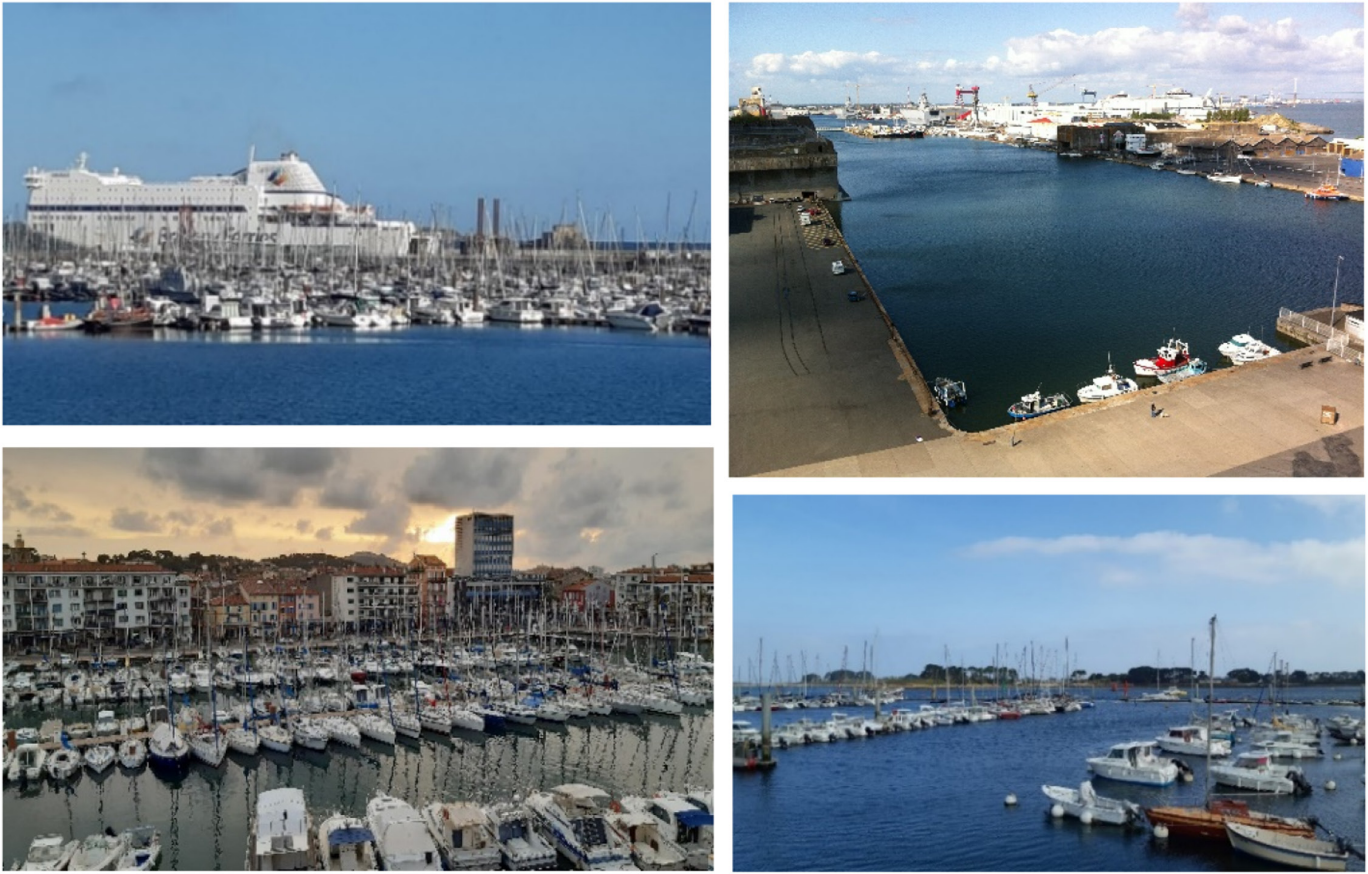

Ports are notably characterized by the accumulation of contaminants (e.g., plastics, pollutants) and specific physical properties (e.g., protection from storms and waves, shading under floating pontoons etc.). They are also often unstable environments (e.g., due to maintenance of the infrastructure). These abiotic features constitute a set of stressors (Table 1) favoring species that can cope with disturbed environment or are tolerant to contaminants (Airoldi et al., 2021; Airoldi & Bulleri, 2011; Figueroa et al., 2021; McKenzie et al., 2012; Rivero et al., 2013). At the landscape level, these hard structures are mostly built‐up on and replace soft‐sediment habitats, thus contributing to composing a mosaic of distinct habitats. Thus, although ports can have locally unique features, they share properties defining a particular type of habitat, characterized by particular species assemblages and forming corridors and networks.

Photo credit: Top right: Ph. Saget—other pictures: F. Viard

Studying evolutionary processes in ports has advantages. First, the size of ports far exceeds that of experimental laboratory infrastructures. Port containment is often strong, with basins closed by locks, which limits exchanges with the outside open sea. While environmental variables (e.g., temperature, salinity, pollutants) cannot be controlled as in the laboratory, they are buffered over large volumes. Second, port habitats are broadly similar to each other, sharing similar anthropogenic stressors (Table 1). Each port thus provides a replicate experiment. Third, because ports are singular habitats, marine organisms encounter artificial substrates that do not exist in nature and are exposed to new chemicals—or at least at unprecedentedly higher concentrations (McKenzie et al., 2012)—and they encounter new assemblages of species, the so‐called biotic environment, often dominated by nonindigenous species (Leclerc et al., 2020). Fourth, the evolutionary processes taking place in ports are occurring over longer times than laboratory experiments but are usually shorter than evolution in natural habitats. Finally, ports constitute a dense network. They are linked to each other through shipping activities across diverse spatial scales (regional with leisure boating up to transoceanic with commercial shipping trade). As shipping activities are diversified (trade, leisure activities, fishing) and ever‐increasing, connectivity among these novel habitats also increases. Ports disrupt previously connected natural habitats but, conversely, promote novel connectivity pathways, resulting in movements of species in and out of their natural range (Aguilera et al., 2020; Bishop et al., 2017; Firth et al., 2016; Henry et al., 2018). These movements are well‐illustrated by nonindigenous species, for which ports are points of entry and facilitate their spread (Dafforn, 2017; Johnston et al., 2017). Ports are nodes of a human‐made network that represents an opportunity for a rendezvous of species, lineages, and genotypes that would not have come into contact naturally, resulting in various scenarios of genetic admixture and adaptation (Geburzi & McCarthy, 2018; Viard et al., 2020).

**TABLE 1:** Examples of abiotic stressors in ports and of their biological effects

Abiotic stressor	Effects	References (examples)
Novel habitat (pontoons, pilings) made of artificial substrates (e.g., steel, concrete) and with particular shape and size	Epibiotic assemblages different from adjacent natural hard substrates (such as stones and rocky reefs); shading effects facilitating the settlement of invertebrate species, such as ascidians, showing a preference for downward surfaces	Connell, 2000; Bax et al., 2002; Rius et al., 2010; Tait et al., 2018
Chemical pollutants (e.g., heavy metals, organic pollutants, polycyclic aromatic hydrocarbons)	Selection for resistance to pollutants; e.g., tolerance to copper enhancing recruitment of the bryozoan *Watersipora subtorquata*; decrease in native species diversity, and dominance by nonindigenous species tolerant to antifouling paints	McKenzie et al., 2012; Piola & Johnston, 2007; Floerl & Inglis, 2005; Tait et al., 2018, and references therein
Artificial light at night (ALAN)	Modification of fish assemblages with night lighting, with an increase in large predatory fish	Becker et al., 2013
Noise pollution	Change in behavior and physiology of invertebrates (e.g., increase oxygen consumption) sensitive to harbors ambient‐noise	Wale et al., 2013
Frequent disturbances due to infrastructure maintenance	High turn‐over of the community (sessile species); Massive die‐off followed by rapid (re)colonization by opportunistic and short‐lived sessile species	Airoldi & Bulleri, 2011; Figueroa et al., 2021; Pineda, Turon, et al., 2016a; Rivero et al., 2013; Ruiz & Hewitt, 2002
Reduced wave actions and tidal currents (physical barriers such as seawalls, breakwater, and jetties)	Reduce currents and flow, favorable to wave action intolerant species; e.g., higher abundance of the seaweed *Codium fragile* on the sheltered side of breakwalls; increased abundance of invertebrates with short‐lived larvae possibly due to larval retention	Bishop et al., 2017 and references therein; Bulleri et al., 2006; Rivero et al., 2013
Ships in abundance	Favor introduction and establishment of nonindigenous species; stowaway pathways for transporting resident port species	Bax et al., 2002; Bishop et al., 2017; Dafforn et al., 2012; Dafforn et al., 2009; Glasby et al., 2007

Embracing a population and evolutionary genetics framework, we here examine the singularities of ports and their interplay with evolutionary processes. We aim to complement Alter et al. (2021) who extensively synthesized adaptive responses and changes in genetic diversity of marine organisms in marine urban environments in general. Here we focus on three interlinked drivers of evolutionary changes related to port characteristics. We first discuss how local and global connectivity reshuffle standing genetic variation within and between ports. We then ask how local adaptation can act in this context and show why it constitutes particularly interesting large‐scale experiments. Lastly, we detail important questions when it comes to anthropogenic hybridization in ports and how they also serve as laboratories for evolutionary studies. From this review documenting evolutionary processes occurring in ports, we finally advocate for establishing ports as model systems for studying evolutionary processes in the Anthropocene.

## 2 PORTS ARE OPENING NEW CORRIDORS FAVORING GENETIC RESHUFFLING

**FIGURE 1:**
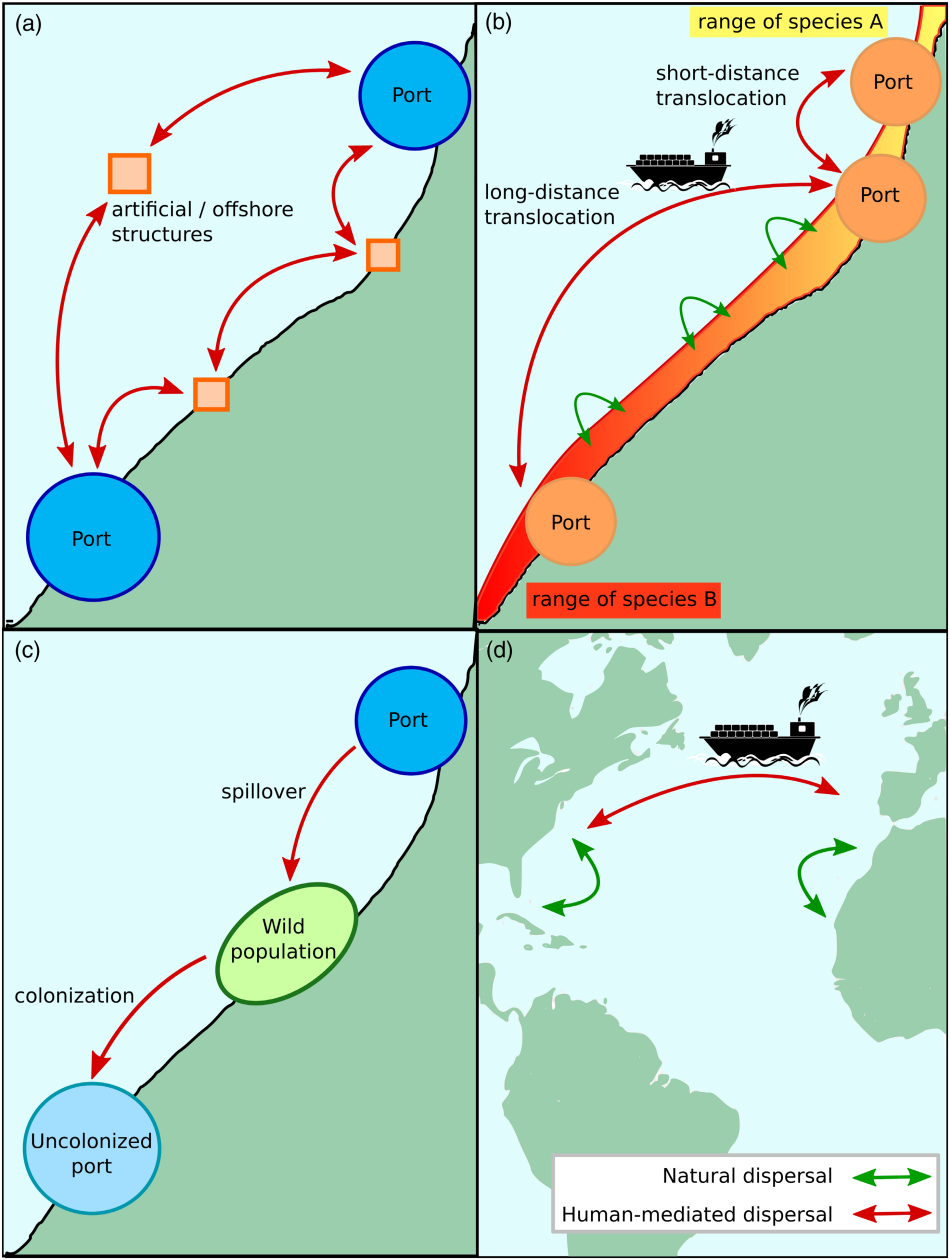
Anthropogenic translocations open new pathways and connect habitats at different scales. (a–c) represent processes happening at a regional scale. (a) Artificial and offshore structures can act as stepping stones and become springboards for organisms to disperse and colonize other locations. (b) Natural dispersal (in green) depends on the species’ dispersal abilities and is mostly done between close locations. Thus, the further two populations are from each other, the more differentiated they will be. Meanwhile, shipping (in red) sustains both short‐ and long‐distance translocations. Dispersal by human action breaks the isolation‐by‐distance patterns and can bring down the genetic structure of populations or make it more complex. (c) Shipping can be responsible for spillovers from ports to wild populations and help organisms colonize locations where they are not yet established. (d) Transoceanic shipping translocates organisms on a global scale, potentially bringing them into contact with geographically distant lineages; some of them might have evolved in complete allopatry.

Human‐driven spread and long‐distance dispersal has been quite well‐documented in artificial habitats including ports. For example, Coolen et al. (2020) showed that the blue mussel *Mytilus edulis* has colonized offshore platforms in the North Sea, beyond the maximum dispersal distance of mussel larvae. Similar stepping‐stone effects have been proposed for explaining the rapid spread of nonindigenous species along the coast, by migration from port to port (Bishop et al., 2017), and to other marine hard infrastructures along the coastline (Airoldi et al., 2015). Such effects are expected to be of particular importance for species with weak natural dispersal abilities, like direct developers or benthopelagic species with a short pelagic phase (<1 day), both expected to have natural dispersal distances below 1 km (Shanks, 2009). One such example is the non‐native tunicate species *Asterocarpa humilis* that brood its larvae up to a late stage. The dense network of marinas along the coast of Southern England might explain the rapid spread (within a 4‐year time frame) of this short disperser via biofouling of adults on leisure boats (Bishop et al., 2015). Ports can facilitate the spread of their resident species, through a stepping‐stone process and a mixture of short‐ and long‐distance dispersal driven by shipping activities in those habitats (Figure 1a,b). Such pathways and processes are expected to have consequences on gene flow, and thus on the distribution of genetic diversity, as shown below.

### 2.1 Ports constitute nodes of an artificial network that is responsible for migration shortcuts

Besides facilitating short‐distance dispersal, shipping also promotes long‐distance migration events that are far exceeding the natural dispersal ability of marine species, as illustrated by the spread of nonindigenous species, with ports forming invasion corridors (Airoldi et al., 2015; Bax et al., 2002; Mineur et al., 2012). These long‐distance dispersals can occur across biogeographic and oceanographic barriers. For example, Bouchemousse et al. (2016) reported the absence of any genetic structure between populations of *Ciona robusta* introduced in Chile and located on both sides of a major and well‐documented biogeographic break at 30–33°S along the coast of Chile. The genetic patterns observed in the introduced range of marine non‐native species are often hard to interpret, because of repeated introductions, high propagule pressure, and secondary human‐driven transports within the introduction range (Viard et al., 2016). These processes are ultimately shuffling the genetic diversity over large scales.

Recently, Hudson et al. (2022) obtained evidence that the timing and the position at which marine invaders enter the world maritime traffic network can make a difference in the resulting genetic structure and diversity observed nowadays. The Asian sea‐squirt *C. robusta* has likely been introduced successively out of its range at least three times, and the introduced populations had time to differentiate and to admix secondarily at some places. Conversely, another tunicate species of Australian origin, *Microcosmus squamiger*, likely entered the worldwide traffic network from a port developed later in the history of maritime trade (i.e., a secondary node). For this species, a uniquely sourced genetic cluster invaded ports worldwide, probably spreading by a stepping‐stone process from port to port.

Regardless of the introduced or native status of the species inhabiting ports, organisms can be transported with no relation to their natural dispersal ability or to hydrodynamic features. Thus for sessile species inhabiting ports, isolation‐by‐distance, or isolation‐by‐currents patterns can be erased. In their study, Lacoursière‐Roussel et al. (2012) showed that the number of trips recorded between pairs of marinas better explained the genetic similarity among populations of the colonial tunicate *Botryllus schlosseri* than did geographic distance. This finding is consistent with the study of Ulman et al. (2019) who examined the nonindigenous species present on the hulls of 600 boats in 25 marinas along the northern Mediterranean coast. They concluded that a large proportion of boats carry nonindigenous species that are often absent in their home port. It is interesting to note that these boats visited on average 7 to 8 ports per year, and some of them sailed from the Eastern Mediterranean to the Western Mediterranean.

**FIGURE 2:**
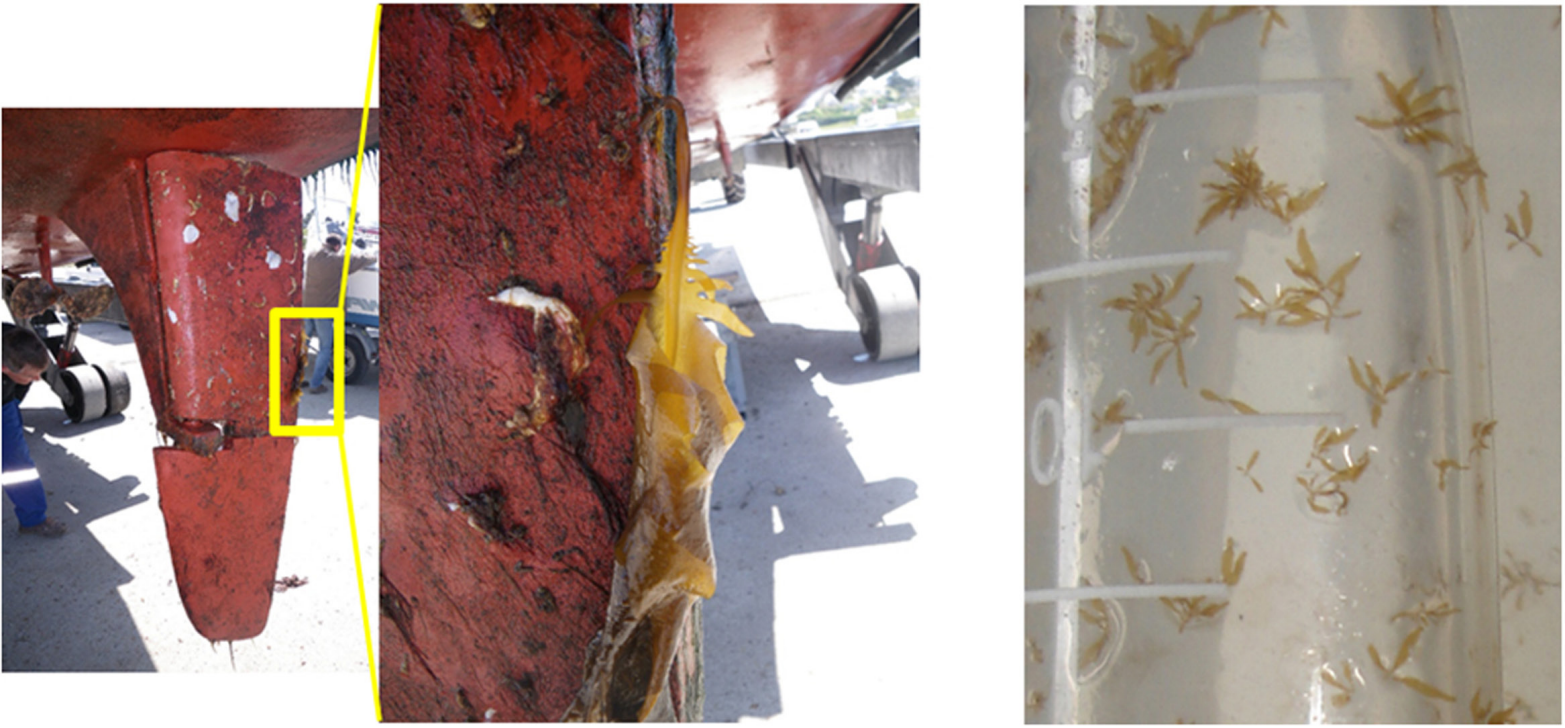
Illustration of the biofouling pathways for spreading the Pacific kelp *Undaria pinnatifida* from port to port. This seaweed native to Asia has been introduced in New Zealand and Europe during the 1970s–1980s. It is a short‐lived species, with a life‐cycle alternating macroscopic diploid sporophytes (left and central picture) and microscopic haploid gametophytes (right picture) that can both be found attached to boat hulls, anchoring systems, or ropes. While natural dispersal by spores or gametes occurs at a very short distance (<10–100 m; Forrest et al., 2000), it can be easily spread over long distance (>100 km) through shipping trade and leisure boating, as evidenced by both field and genetic studies (Epstein & Smale, 2017; Guzinski et al., 2018; South et al., 2017). Ports, and associated shipping and boating, provide major expansion pathways and are responsible for long‐distance dispersal events of this introduced seaweed.

Organisms can be transported on ship hulls at various stages of their life cycle, including microscopic stages, such as postlarval/juvenile stages for invertebrates or gametophytes in seaweeds. This connectivity driver can create a mosaic genetic structure. This is exemplified by the Pacific kelp *Undaria pinnatifida*, which has limited natural dispersal ability but can be easily transported on anchors, ropes, and hulls (Figure 2). In Brittany, where this seaweed is largely distributed, and particularly conspicuous in marinas, a SNP‐based study revealed a patchy genetic structure most likely explained by anthropogenic transport in and out of ports (here marinas), inducing low levels of differentiation either between distant or close locations (Guzinski et al., 2018). Similar patterns of mosaic genetic structure unrelated to distance, and including long‐distance dispersal events related to boating, have been reported for other species with short‐lived larvae, such as the sea‐squirt *Ciona intestinalis* in its native range in the English Channel (Hudson et al., 2016), the cosmopolitan and cryptogenic tunicate *B. schlosseri* in Canada (Lacoursière‐Roussel et al., 2012) or the colonial invasive tunicate *Didemnum vexillum* (Prentice et al., 2021). We note that exceptions to this observation exist, such as the spread of the ascidian *Styela plicata* in harbors along the Spanish coasts (Pineda, Lorente, et al., 2016b). However, altogether, human‐mediated pathways lead to connection shortcuts between distant populations and facilitate the shuffling of genetic diversity and the mixing between genetic lineages of species inhabiting ports.

### 2.2 Gene flow spillover from ports to natural habitats

Most of the genetic studies comparing populations from natural and port habitats have been carried out on nonindigenous species (but see Fauvelot et al., 2009, described below), to examine their expansion and risk of spread into natural habitats. These studies have most often documented gene flow between the port and natural habitats (Figure 1c). Spillover effects, which is the seeding of natural habitats by dispersers from populations established in ports, have been for instance shown for the introduced Pacific kelp *U. pinnatifida* (Guzinski et al., 2018). This spillover process is similar to escapees of cultivated marine species from aquaculture farms, leading to deviations from natural dispersal patterns (e.g., goldsinny wrasse fish; Jansson et al., 2017) and population reinforcement at species edge range (e.g., corkwing wrasse; Faust et al., 2021). Similarly, in the case of the Pacific kelp, ports have been shown to promote the establishment of this introduced seaweed in the wild (Epstein & Smale, 2018a,b; Guzinski et al., 2018). Interestingly, spillback events, i.e., the colonization of novel ports by individuals coming from the wild, have also been documented in *U. pinnatifida* (Salamon et al., 2020), suggesting regular bi‐directional gene flow between wild and artificial habitats. Likewise, for the ascidian species *Microcosmus squamige*r, Ordóñez et al. (2013) found no differences between populations on natural and artificial substrates, suggesting regular exchanges between the two populations categories.

Ports may influence the genetic diversity of populations in natural habitats because of sustained immigration from port populations to neighboring wild populations. Using a paired replicated sampling design, a microsatellite‐based genetic analysis of the limpet *Patella caerulea* showed that populations established in artificial habitats, breakwaters, have lower genetic diversity than populations established in nearby natural habitats, reefs, while not being genetically differentiated (Fauvelot et al., 2009). Based on these results, the authors suggested that the expanding populations from artificial habitats might lead to a decrease in the overall genetic diversity of the study species on a regional scale. Studies are still too scarce for assessing the true importance and consequences of the influence of port‐to‐wild gene flow. However, ports are without doubt opening novel pathways, and they may act as sources for unstable or endangered natural populations or conversely be sinks due to propagule retention in ports. These outcomes are likely dependent on specific properties, such as local population density, reproductive outputs, biofouling abilities, or pelagic larval duration. There is thus a dire need for more studies of species inhabiting the two types of habitats in the same region, to provide a comprehensive understanding of the impact of these novel habitats on the eco‐evolutionary dynamics of the biota in natural habitats, notably in terms of the spread of advantageous alleles or, conversely, migration load and “genetic pollution” as discussed below.

## 3 PORTS AS NATURAL EXPERIMENTS OF ADAPTIVE EVOLUTION IN A PATCHY ENVIRONMENT

Ports are singular habitats that can select for particular genotypes (Box 1, Table 1). The network of urbanized islands (i.e., ports) connected by anthropogenic gateways (i.e., maritime traffic), intertwined in a sea of wild habitats (Figure 1b,c) is the ideal place for convergent evolutionary changes (Alter et al., 2021; Santangelo et al., 2022). We will first examine how adaptation originates and propagates. At the gene level, locally adapted alleles can (i) have multiple independent mutational origins, (ii) be ancestrally shared, or (iii) spread throughout subpopulations via gene flow, or a combination of these three scenarios (Bierne et al., 2013; Welch & Jiggins, 2014). We then discuss possible outcomes of local adaptation in ports once they have evolved. Importantly, can such adaptive changes resist gene swamping (i.e., can locally advantageous alleles persist in the population)? Or conversely can they be exported into the wild? Or finally, can the coupling between new port adaptation and a nonindigenous genetic lineage favors the establishment and spread of this nonindigenous lineage that otherwise would be trapped in its native range by natural barriers? We address these two issues in turn.

### 3.1 Parallel adaptation in a patchy environment: Mutation, migration, or shared ancestral variation?

A population faced with a new human‐induced selective pressure can only adapt if the appropriate genetic variation is available. This genetic variation might (i) stem from new mutations, or (ii) already segregate in the population as a standing genetic variation, or (iii) come from gene exchange with other populations or species. Understanding the relative importance of these sources of adaptive variation has practical implications for conservation, biological control, and infectious disease prevention (Harpak et al., 2021; North et al., 2021; Pennings, 2012). We only have a few examples of convergent adaptation to ports, but the most compelling example, in killifish (*Fundulus sp*.), illustrates very well that all three sources of genetic variation can be observed in a single study system, and often in combination.

**FIGURE 3:**
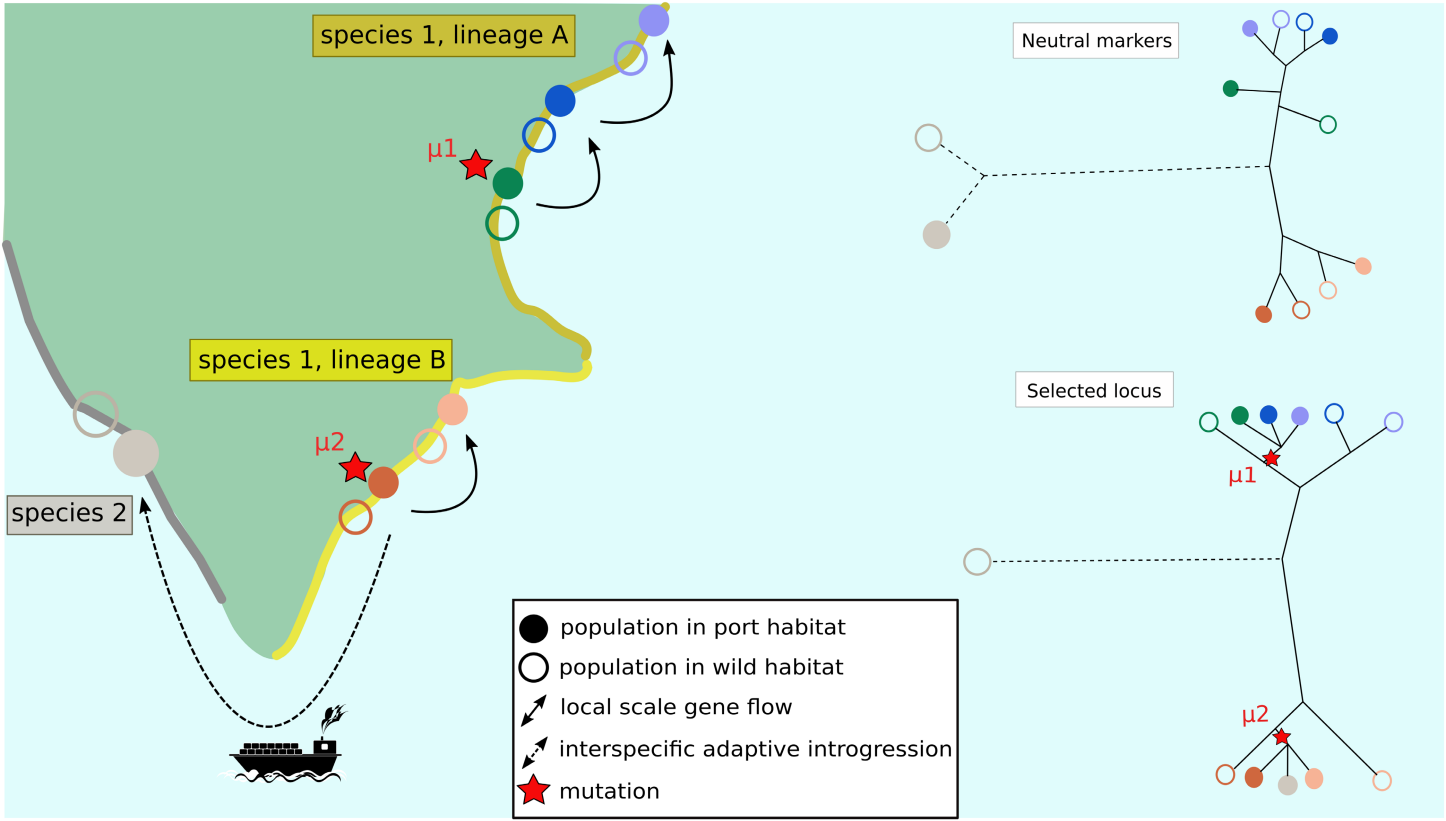
Adaptation in a patchy environment. In this schematic scenario, two lineages of one species (species 1) are separated by a barrier to gene flow. In each location, one population is found in a port habitat (filled circle), another one in a wild habitat (empty circle). Two independent convergent mutations (μ1 and μ2) related to adaptation to the port environment appear in one population of each lineage (adaptation by de novo mutations). These mutations then propagate to close populations found in the same port habitat by gene flow through wild populations (thanks to migration‐selection balance that maintains a low frequency of port‐adapted alleles in wild populations, aka transporter hypothesis) or helped by maritime traffic. This latter anthropogenic pathway may introduce individuals with the mutation to an area where a second species (species 2) is found in port habitats. Introgression from the introduced species to the second species occurs, as this mutation is advantageous in the port environment. This process is called adaptive introgression. On the right of the figure, the upper tree shows the genetic relationships at neutral markers between the different populations involved, while the second tree is obtained with the selected locus.

Killifishes are small fish living on the East coast of North America. These fishes provide us with a beautiful example of adaptation to lethal levels of industrial pollutants in ports. To adapt, different populations and species have followed different evolutionary paths but most often target the same or similar genes, suggesting strong adaptive constraints or low genetic redundancy. Reid et al. (2016) studied populations of the Atlantic killifish, *Fundulus heteroclitus*. They sequenced 384 whole genomes from four pairs of pollutant‐tolerant and pollutant‐sensitive populations along the US Atlantic coast (Figure 3). The general genome‐wide pattern confirmed two lineages across a phylogeographic break centered in New Jersey (Duvernell et al., 2008). They identified candidate genomic regions for pollution resistance with a window‐based *F*_ST_‐outlier approach. Although most candidates were specific to a single tolerant population, the top‐ranked outliers were shared among some populations and contained genes of the aryl hydrocarbon receptor (AHR) pathway involved in the protection from hydrocarbon toxicity. The aryl hydrocarbon receptor‐interacting protein (AIP) gene showed the highest levels of differentiation among all four pairs of tolerant/sensitive populations. However, a different haplotype has swept in the northern and the southern lineages of the species. These results support repeated adaptation from de novo variants targeting the same genes, as expected for a truly new environment never encountered by other populations of the species before (contrary to, e.g., freshwater in sticklebacks (Jones et al., 2012), wave action, and crab predation in *Littorina* (Johannesson et al., 2017) or coastal habitat in bottlenose dolphins (Louis et al., 2021)), and for a highly constrained trait with little genetic redundancy. However, at a local scale within a lineage, adaptive variants were shared. Lee and Coop (2017) reanalyzed the data by fitting alternative adaptive scenarios. They confirmed independent sweeps in the two lineages at the AIP gene and found support for the three northern populations sharing the same beneficial allele, either via migration or selection on a young standing variant. The latter two scenarios are incredibly difficult to discriminate as they produce very similar footprints (Bierne et al., 2013) and even sophisticated methods, such as the one of Lee and Coop (2019), struggle when populations are highly related. The study of another species, *F. grandis*, provided a new look at the issue. Oziolor et al. (2019) searched for signatures of selection that co‐vary with a pollution gradient in the extremely polluted Houston harbor, well known for its petrochemical industry and dedicated seaport. They again found that genomic regions showing the strongest signatures of selection contain genes of the AHR pathway. A region containing an AHR deletion was surprisingly more similar to *F. heteroclitus* haplotypes than to other *F. grandis* haplotypes. Using the Lee and Coop method, Oziolor et al. (2019) demonstrated that introgression of the deletion‐bearing haplotype was much more likely than a shared ancestral polymorphism. Given that *F. heteroclitus* does not live in the Gulf of Mexico, introgression was likely mediated by recent human‐assisted transport. This is one of the best examples of adaptive introgression mediated by human activities to date.

### 3.2 Migration load, gene pollution, or new lineage escape

Although rarely examined, several studies have documented gene flow between natural and port populations (see Section 2). Looking for adaptive responses specific to ports thus also requires considering neighboring wild populations and the possible threat that port populations can impose on them. There are three broad possible outcomes:

1. Given that ports are small pockets of urbanized habitats and many marine species have high dispersal potential, gene swamping may prevent local adaptation from evolving in ports if the selection is not strong enough (Lenormand, 2002), or if genomic architecture (i.e., the genomic location of alleles contributing to the adaptive trait) does not evolve in concert to protect locally adapted gene regions from swamping (Schaal et al., 2022; Yeaman, 2013, 2015). Although the killifish study suggested adaptation to the port could be fast‐paced, some other works have failed to confirm evidence for local adaptation. For example, Guzinski et al. (2018) did not find evidence of local adaptation between ports and natural rocky habitats colonized by the brown nonindigenous alga *U. pinnatifida*, which may be due to high gene flow (see Section 2.2; Figure 1c). One alternative explanation for gene swamping is that the density of molecular markers was too low to pinpoint selection targets. To date, only very few studies have used whole‐genome sequencing to study adaptation to ports. The open nature of small ports like marinas and fishing ports could favor local gene swamping. While, available evidence suggests limited connectivity between large ports and the local seaside because of the presence of docks and locks in these large ports. Controlling this connectivity could be an unconsidered management option for ports to limit the establishment of locally adapted genotypes or species. Usually, ports develop by increasingly enclosing water masses with new docks and locks, especially in the most enclosed part of the port. Deliberate opening up of ports could favor the entrance of seaside waters and propagules of native genotypes or species in ports, which could result in such a swamping of locally adapted port lineages. This evolution‐aware transient open‐port strategy could be worthy of consideration in port management plans for biodiversity.

2 Once a locally adapted genotype or lineage is established in a port, one may fear gene flow toward surrounding natural populations (uncontrolled flow of detrimental, locally adapted alleles, or genetic incompatibilities into wild populations). Species that manage to colonize ports often make dense luxuriant populations. Therefore, the potential spillover pressure of port‐ecosystems toward surrounding populations, as shown for *U. pinnatifida* (Epstein & Smale, 2018a,b; Guzinski et al., 2018; see Section 2.2) should be examined. This is of concern because ports are sometimes considered as a putative refuge for species coping with global change and anthropogenic pressure. They are even proposed as a solution for mitigating environmental impacts and contributing to the management and protection of marine coastal ecosystems, notably through eco‐engineering approaches (Mayer‐Pinto et al., 2017). Given adaptive trade‐offs are common, it is likely that port adaptations should be costly in the wild. Gene knockdown alleles found in killifish populations of the pollution gradient in Houston harbor are an informative example of this trade‐off (Oziolor et al., 2019). We also suspect that, although luxuriant, port populations are nonetheless small pocket populations that might accumulate deleterious mutations faster than wild populations that, although less dense, are broadly and continuously distributed. While there is little experimental and theoretical evidence to confirm or refute the hypothesis that maladaptive gene flow from portuarized to wild populations is a real concern for conservation, there is no doubt that this hypothesis deserves further empirical evidence, and as such is an important direction for future research.
3 Finally, the last threat could be the escape of new portuarized lineages out of the port, where entire genomes instead of some alleles would spread into the wild. This scenario implies that the portuarized population is a differentiated (semi‐)isolated lineage. It should thus likely require either long‐term evolution, or admixture and (un)coupling with pre‐existing semi‐isolated lineages introduced in ports by the shipping traffic (see below). If the conditions are met, human‐altered evolution in port‐ecosystems can result in super lineages (i.e., intrinsically fitter) that can benefit from genetic (heterosis) and demographic (density gradient) boosters favoring escape and spread outside of ports. Despite a lot of evidence of invasive marine species entering through ports and spreading from their entrance point, there is little evidence of the same thing occurring for a lineage (but see a dock mussel ecotype that escaped from the port of Brest to the neighboring estuary in Simon et al., 2020, as detailed in Section 4 below). However, we expect forthcoming genome‐wide surveys will unmask such hidden invading lineages in the near future.

## 4 PORTS AS IDEAL ARENAS FOR THE STUDY OF ANTHROPOGENIC HYBRIDIZATION

Maritime traffic associated with ports has opened secondary contact of species across biogeographic boundaries (Figure 1d; Sardain et al., 2019). Admixture—the genetic mixing of differentiated taxa—is considered as one possible way by which introduced species may adapt to their novel environment, i.e., through recombination processes leading to evolutionary novelties (for reviews see, Bock et al., 2015; Rius & Darling, 2014; Rius et al., 2015). Admixture can happen along the genetic gradient of differentiation of the speciation continuum (Roux et al., 2016). The term admixture can be used to describe both intra‐ and interspecific genetic mixtures. The term hybridization is exclusively used to characterize the process of interbreeding between partially reproductively isolated lineages and leading to admixture. Besides admixture between different populations of a given species, another evolutionary consequence of transports of species by shipping is anthropogenic hybridization—hybridization driven by human‐mediated changes through environment alterations or species displacement (Viard et al., 2020).

We focus in this section on interspecific hybridization and exclude the discussion of the introduction and admixture of multiple sources of the same species. The role of human disturbance in hybridization has been well studied in plants and numerous works have shown that the contribution of nonindigenous species is non‐negligible in the production of hybrids (Abbott, 1992; Anderson & Stebbins, 1954; Guo, 2014; Preston & Pearman, 2015). It is thus expected that the marine realm, and ports more specifically, should set the scene for rampant anthropogenic hybridization. Despite this expectation, the low amount of observed and studied examples represents a paradox. In this section, after considering the potential causes of this paradox and how to overcome it, we discuss the population genetic outcomes of anthropogenic hybridization in ports and how ports can serve as study systems for speciation research.

### 4.1 Detection and study of anthropogenic hybridization in and around ports

While anthropogenic hybridization in ports can be thought to be frequent given the recurrent introduction of diverse nonindigenous species, studied examples remain particularly scarce (Le Moan et al., 2021; Popovic et al., 2020; Simon et al., 2020). It is currently difficult to evaluate if the low number of anthropogenic hybridization examples in ports have a biological basis, or whether it is simply a by‐product of a blind spot of research pertaining to the field of marine urban evolution. Two main issues might hinder the detection of anthropogenic hybridization in ports, explained below.

The easiest cases of hybridization to notice are those where two species have easily identifiable morphological traits. However, cryptic species—evolutionary divergent lineages that are indistinguishable based on conspicuous morphological characters—are particularly abundant in the marine environment (Appeltans et al., 2012; Chenuil et al., 2019). Therefore, the exclusive use of morphological classification in the search for nonindigenous species in ports might bias our perceived landscape of species in contact. Chenuil et al. (2019) proposed two nonexclusive causes for the amount of marine cryptic species: (i) Around half of the cryptic species still require taxonomic revision, and (ii) life‐history traits of marine species could explain their propensity to have cryptic species (e.g., large population sizes or high within‐species morphological variation). Overall, as described by Pante et al. (2015), the failure to describe and recognize evolutionary‐relevant lineages can create erroneous starting hypotheses on which downstream analyses are based (in their case, connectivity).

Current DNA‐based methods used for describing communities and detecting nonindigenous species are not equipped to detect hybridization. Revealing hybridization is a difficult task that requires the use of specific DNA‐based methods (Payseur & Rieseberg, 2016; Viard & Comtet, 2015). While metabarcoding studies are important for the discovery of invasions, they suffer from a lack of power to investigate hybridization, which typically requires multilocus analysis (e.g., Le Moan et al., 2021; Simon et al., 2020). Two cases can be considered where hybridization cannot be demonstrated in the data when using a limited number (usually one or two) of barcoding markers: (i) The locus used for barcoding is introgressed from the native species into the nonindigenous species population used as a reference and/or the hybrids; (ii) the locus used is not discriminant enough at the species complex scale. Both examples could easily happen for species still in the gray zone of speciation. For instance, *Mytilus trossulus* was initially spuriously identified in an eDNA study in an area with natural *M. edulis*, using a mitochondrial marker. This was due to the fact that the reference samples of *M. trossulus* in the database come from the Baltic sea, where the *M. edulis* female mitochondrion has introgressed (Couton et al., 2022). We nonetheless expect that progress will allow us in the near future to better and more systematically identify cryptic hybridization, with specimen sampling first but also with dedicated eDNA sample analyses as a second step.

Finally, when introductions can be properly identified, determining the hybridization status of nonindigenous species and follow‐up detailed studies require the use of genomic or at least multi‐marker methods. Le Moan et al. (2021) provided a compelling example of introgression that could have been missed by only looking at a few markers along the genome. By using a genome‐wide method (ddRAD sequencing), they uncovered an introgression breakthrough from *C. robusta* into *C. intestinalis* in a specific genomic hotspot, which was observed in multiple zones of contact in ports between the two species. This recent introgression was previously missed even with the use of a large ancestry informative SNP panel, due to its very localized position in the genome (Bouchemousse et al., 2016; Le Moan et al., 2021). The analysis of whole‐genome sequences provided support that this introgression breakthrough is adaptive (Fraïsse et al., 2022).

### 4.2 Port characteristics provide suitable conditions to study reproductive isolation

Evolutionary biologists are still far from understanding all the subtle nuts and bolts controlling the outcomes of hybridization, both theoretically and empirically (but see Abbott et al., 2013; Abbott et al., 2016, for reviews on the subject). On par with natural secondary contacts, anthropogenic hybridizations provide “laboratories for evolutionary studies” (Grabenstein & Taylor, 2018; Harrison, 1990; Hewitt, 1988; McFarlane & Pemberton, 2019). Recent secondary contacts in ports provide evolutionary biologists with in situ laboratories that are quite different from postglacial hybrid zones. Contrary to postglacial contacts, anthropogenic contacts are more recent, with fewer generations of admixture, and in nonequilibrium situations (spreading waves). Additionally, the demography is different, with more asymmetry between the two lineages in contact, and, above all, contacts are often replicated at several places while postglacial hybrid zones have little replication. Viard et al. (2020) already pinpointed several evolutionary questions related to anthropogenic hybridization in marine environments. We are particularly interested here in how the study of such contacts in ports could advance research in evolutionary biology.

The outcome of anthropogenic hybridization, similarly to natural secondary contacts, is dependent on various factors including reproductive isolation mechanisms in place between the species in contact, the environment where the contact takes place, and the demographic context (Abbott et al., 2013; Viard et al., 2020). Factors influencing hybridization that are specific to ports include, for example, changes in reproductive barriers, new environments, variability in propagule pressure (Viard et al., 2016), or enclosed spaces (see Section 3).

Anthropogenic displacement potentially brings together geographically distant lineages that might have evolved in complete allopatry. In such cases, given a similar divergence, species are expected to show reduced prezygotic reproductive isolation compared with species evolving in sympatry (Coyne & Orr, 1997; Matute & Cooper, 2021). Conversely, species evolving in parapatry or with a history of recurrent secondary contacts might present increased prezygotic isolation due to reinforcement processes (Servedio & Noor, 2003). While reinforcement has been shown in multiple groups, evidence of this process is still rare among marine taxa (Palumbi, 1994). Incidentally, hybridization is expected to be easier between species naturally separated by biogeographic barriers forced into contact by human‐mediated displacement. Geographic barriers are the first reproductive barrier to be broken down by ports and associated anthropogenic activities (Figure 1d).

Ports can secondarily provide environments disrupting pre‐ and postzygotic barriers to gene flow. For instance, turbidity caused by eutrophication or increased suspended sediment is known to impact mate choice in aquatic environments. While the consequences have mainly been studied in freshwater fishes (Candolin et al., 2007; Seehausen et al., 1997, 2008), marine fishes could be exposed to the same constraints in ports (Järvenpää & Lindström, 2004; Todd et al., 2019). Additionally, chemical cues for sexual recognition, which exist in marine organisms in diverse phyla (Evans et al., 2012; Hay, 2009), could also be disrupted in port environments due to xenobiotic molecules. Finally, urban environments create new habitats and postzygotic selection pressures (Alter et al., 2021; Todd et al., 2019), thereby opening selective potentials for hybrids that might display intermediate or transgressive phenotypes (Anderson & Stebbins, 1954; Bell & Travis, 2005; Rieseberg et al., 1999, 2007). In other words, natural fitness landscapes that previously restricted gene flow between two species could be modified by urban stressors.

In addition to environmental conditions, demography is influencing the outcome of anthropogenic hybridization. In ports continuously connected to other areas of the planet, the propagule pressure might be substantial and steady (Viard et al., 2016). We thus expect that increased propagule pressure will reduce the founder effect that small introduced populations usually endure. Combined with a general preponderance of nonindigenous species in ports, it is likely that the population size of the nonindigenous species equals or surpasses the native one locally. During the invasion, introgression is predicted to occur from the native to the introduced species due to asymmetric population size in favor of the native in the invasion front wave (Currat et al., 2008). If large populations of nonindigenous species are established in ports, the prediction could shift, with introgression occurring from the nonindigenous species to the native species (Viard et al., 2020), as shown with the introgression of the tunicate *Ciona intestinalis* by its introduced congener *C. robusta* (Le Moan et al., 2021). Estimation of the impact of propagule pressure variation on the outcome of anthropogenic hybridization is lacking, as might be expected due to the complexity of accounting for all the interacting parameters of such scenarios. Advancing our understanding of this issue will require the integration of knowledge in speciation research and invasion science.

In terrestrial urban evolution studies, different cities are efficiently used as repeated experiments of local adaptation (Santangelo et al., 2020, 2022). The presence of multiple interconnected ports is also a powerful replicated testing ground, as in the example of dock mussels (Simon et al., 2020). Hybridization was observed in French ports on the Atlantic Ocean and the English Channel, where the introduced Mediterranean *Mytilus galloprovincialis* hybridized with native *M. edulis* (Simon et al., 2020). Dock mussels form homogeneous admixed populations established in different ports of the English Channel and Atlantic Ocean, providing large‐scale mesocosm‐like replicates for the future outcome of this anthropogenic hybridization. The similarity in admixture suggests the two species got admixed in one location and then dispersed to other ports by human‐mediated transport. Additionally, as biotic and abiotic environments slightly vary between ports, it could open the possibility to disentangle the different factors important for the maintenance of those hybrid swarms. For instance, dock mussel populations are confronted with different native genetic backgrounds. One striking pattern emerging from dock mussels is the sharp clines in allele frequencies present at the entry of ports between dock mussels and native mussels, which constitute repeated small‐scale hybrid zones. The hybrid zones that can be maintained at the entry of ports are interesting marine case studies for urban evolutionary biology. Two factors could play major roles in the maintenance of the created population structure: (i) In a classic tension zone model where clines are maintained by postzygotic selection on hybrids, the decrease in gene flow at the entry could be enough to trap a hybrid zone (Barton & Hewitt, 1985), and (ii) the environmental difference between ports and the natural habitat could create coupling between local adaptation loci and the rest of the genome (see Section 4.3 below).

### 4.3 Diverse outcomes

Outcomes of hybridizations have been described extensively both in natural (e.g., Abbott et al., 2013; Edelman & Mallet, 2021; Moran et al., 2021) and anthropogenic settings (e.g., Grabenstein & Taylor, 2018; McFarlane & Pemberton, 2019; Ottenburghs, 2021). The transient and final result of hybridization between two lineages is strongly dependent on the divergence between them and existing reproductive isolation, and on the demographic context of the secondary contact. The processes of pre‐ and postzygotic isolation and recombination shape the genomic patterns of ancestry that can be found in hybrid zones (either naturally produced or anthropogenic). As a simplified overview, if hybridization is limited to the production of first‐generation hybrids (F1), this represents an evolutionary dead end and results in a waste of reproductive outputs for parental lineages. When F1s are fertile, hybridization can lead to scenarios going from a complete mixture of the two parental genomes, more or less homogeneous along the genome, to highly restricted introgression only impacting a small part of the genome (e.g., Le Moan et al., 2021). Just as it is done for natural hybrid zones, genome‐scale studies are required to understand the architecture of reproductive isolation in port hybrid zones.

Anthropogenic hybridization in ports can lead to the emergence of new portuarized lineages or what could sometimes be called hybrid species (Mallet, 2007; Schumer et al., 2014). Hybrid speciation can happen either through allopolyploidization or by a stable ploidy recombinatorial process (homoploid hybrid speciation; Mallet, 2007). As highlighted in the previous section, new genomic combinations can produce transgressive phenotypes that might be readily suited to the port environment (Mallet, 2007). The case of dock mussels (see Section 4.2), being a homogeneously admixed lineage stably conserved between different ports, raises the question of its status. While the hybrid character of this portuarized *Mytilus* lineage has been demonstrated, the reproductive hybridization‐derived isolation is still to be determined according to Schumer et al. (2014) criteria, including reproductive isolation between dock mussels and the Meditteranean *M. galloprovincialis* parental lineage.

We postulate that coupling between local adaptation clines and intrinsic isolation clines could be a powerful process limiting the spread of an invasive genomic background outside ports (Box 2). This scenario may be readily happening in dock mussels at the entry of ports. The hybrid swarms formed by dock mussels are thought to have reshuffled reproductive incompatibility loci that could still maintain the separation with native mussels (Simon et al., 2020, 2021). This process might explain the lack of invasion of natural habitats by dock mussels, in contrast with examples of rapid invasion by *M. galloprovincialis* in areas without much hybridization (Saarman & Pogson, 2015) or without native *Mytilus* congeners (Branch & Steffani, 2004). Interestingly, dock mussels are known to have escaped the port environment in the instance of the Bay of Brest (Simon et al., 2020). It is difficult at this point to conclude if this outcome is due to the specific environment (e.g., the presence of an estuary close to a port environment), a reduced reproductive isolation with the local *M. galloprovincialis* inducing weaker coupling, or a phase reversal of the coupling between exogenous and endogenous backgrounds (Bierne et al., 2011). In this latter case, one might predict that *M. galloprovincialis*, if it is confirmed to really be intrinsically fitter than other species/lineages (as hypothesized in Bierne et al., 2006), can take advantage of ports as gateways for colonizing new areas that would be unattainable otherwise.

BOX 2 **Ports and the (un)coupling hypothesis**

The coupling hypothesis postulates that genetic barriers between pre‐existing semi‐isolated lineages can sometimes couple with new environmental heterogeneities (Abbott et al., 2013; Bierne et al., 2011). Under this hypothesis, the distribution of lineages according to the environmental landscape can be new, although the genetic barriers that maintain genetic divergence between lineages are old. Under this model, genotype‐environment associations are better explained by pre‐existing intrinsic reproductive barriers that became trapped by an ecotone, just like it could easily be trapped by local dispersal barriers or density troughs (Barton & Hewitt, 1985; Bierne et al., 2011).

We can hypothesize that port entries might act as environmental and/or dispersal barriers capable of trapping pre‐existing reproductive barriers (Figure step 3). In contrast with their preponderant role in the influx of alien species, ports could also act as retention basins allowing to slow the spread of an invasive background if endogenous barrier loci can stay coupled with exogenous barriers at the entry of the port. This hypothesis will require the acquisition of evidence of both environmental data, and examples of anthropogenic hybridizations trapped in ports (Simon et al., 2020). We hope future studies of anthropogenic hybridization in ports will be vigilant to not neglect the coupling hypothesis when detecting genotype‐environment associations at the entry of ports. Indeed, local adaptation to port environments might explain the position of genetic clines, but endogenous barrier loci might be the main factor that maintains them.

Conversely, however, ports can also promote the spread of introduced semi‐isolated lineages if connectivity, environmental heterogeneity, and population densities favor new associations between local adaptation genes and intrinsic barriers. Once a semi‐isolated lineage has managed to colonize a port, the concomitant roles of high propagule pressure, low population density of the local lineage in the port, and local selection can result in the escape of such lineage from the port environment. As a result, a recombinant genotype that associates the fittest lineage with wild‐adapted alleles reaches a sufficient frequency to initiate a new wave of advance in the natural environment (Figure step 4).

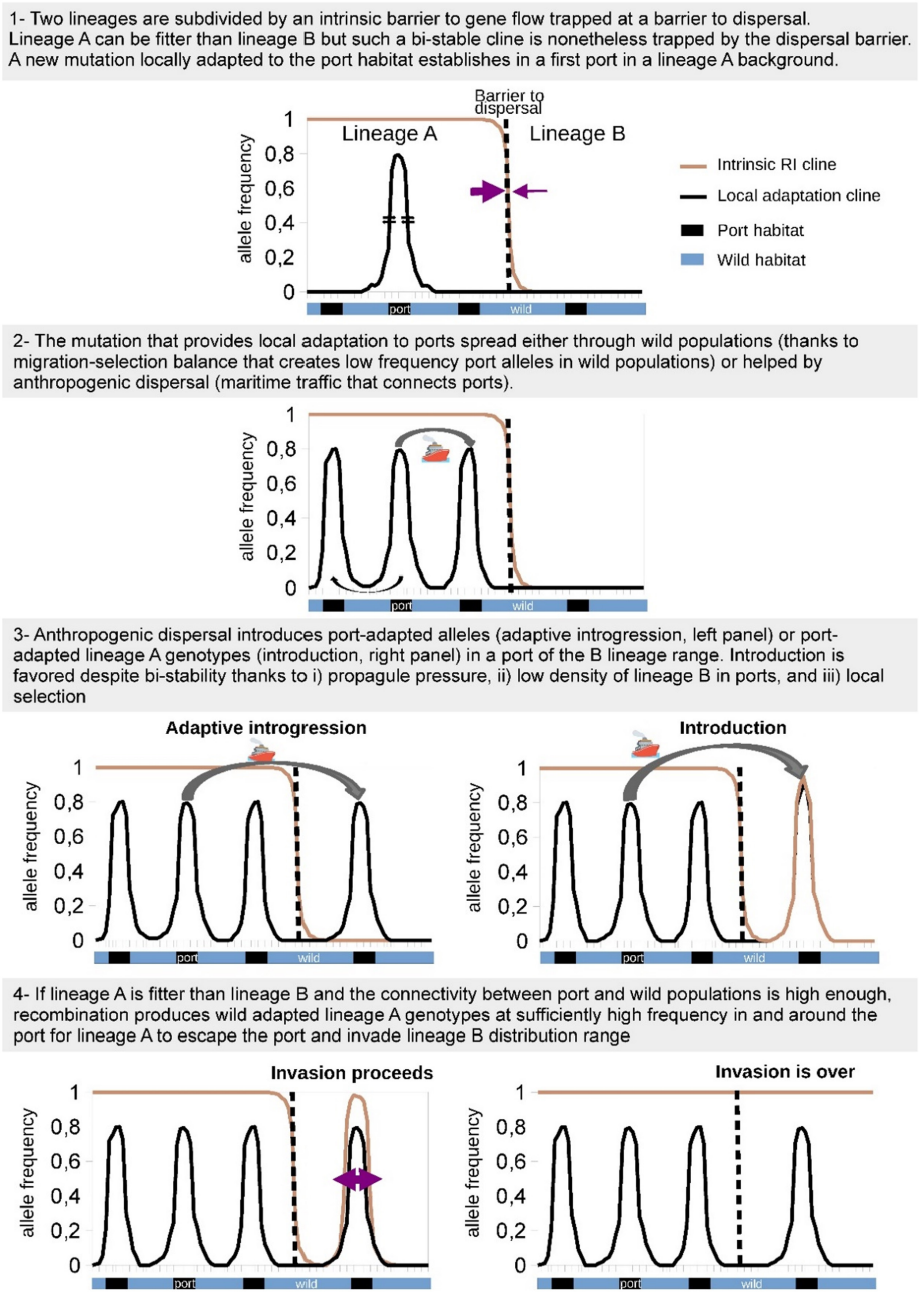

## 5 PORTS AS MEANINGFUL URBAN PLAYGROUNDS FOR EVOLUTION STUDIES

**FIGURE 4:**
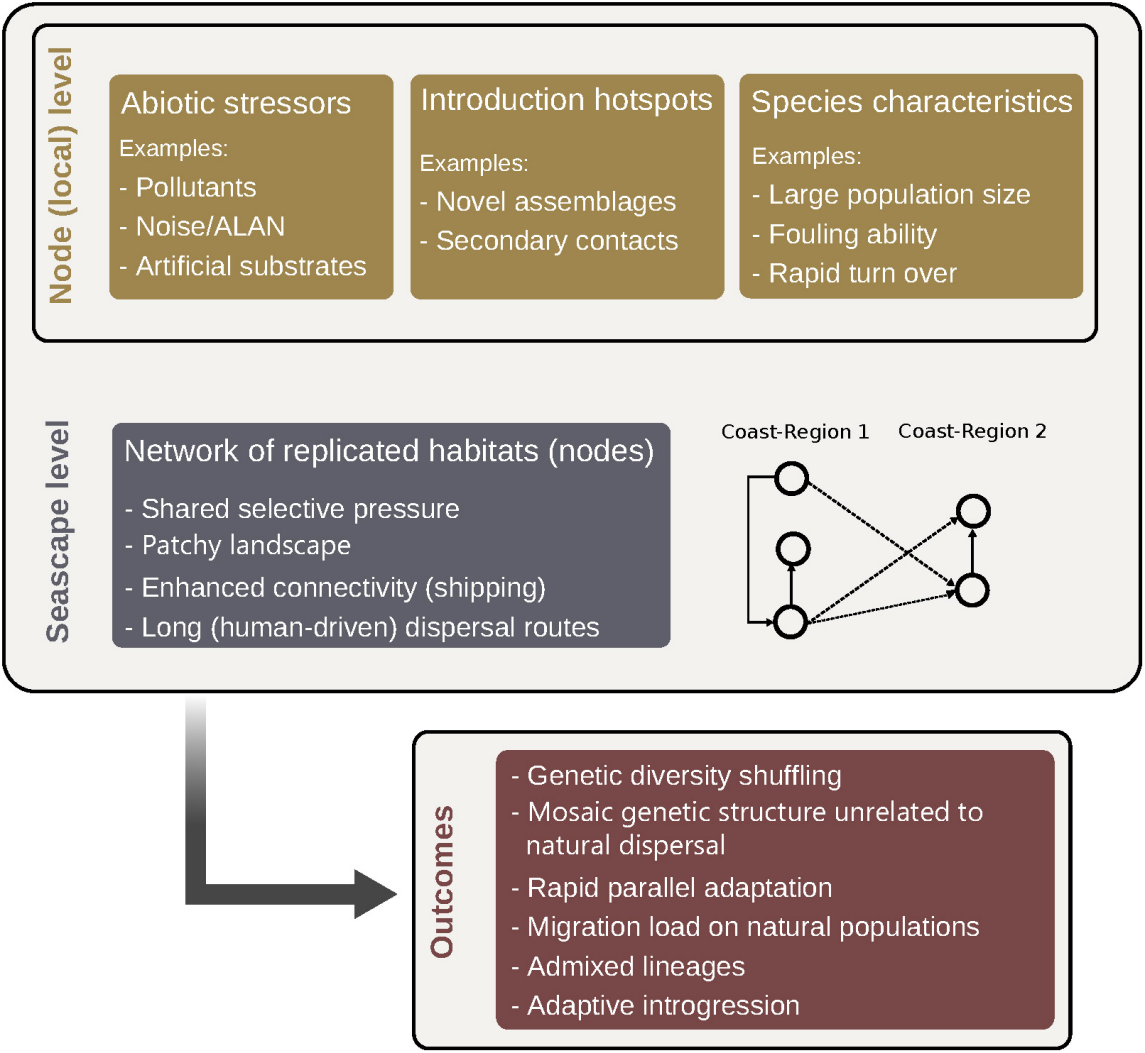
Biological portuarization and its evolutionary outcomes. Ports are singular habitats due to their particular abiotic and biotic properties, at local and global (seascape) levels. They are the port‐of‐entry of non‐native lineages and species and the nodes of a vast and dense network. Evolutionary outcomes already documented are diverse, including genetic diversity shuffling, rapid adaptation, putative risks associated with gene flow in natural habitats, admixture, and hybridization among others.

Ports constitute meaningful urban evolutionary playgrounds where anthropogenic pressures and their effects on species lead to what we propose to call and define “biological portuarization” (Box 3). Anthropogenic activities drive evolutionary changes in response to disturbed and changing environments, and this is well‐exemplified in ports. In previous sections, we documented a series of evolutionary outcomes linked to port singularities, as summarized in Figure 4. We have shown that the connectivity provided by human activities in ports—both at regional and global scales—combined with urban stressors, provide fertile ground for evolutionary processes. Indeed, both the reshuffling of genetic diversity and the selection of port environments can be the source of local adaptations. Additionally, anthropogenic hybridization is expected to be an important phenomenon in ports and provide a new avenue for the study of reproductive isolation and the outcome of hybridization. Other evolutionary consequences also start to be documented, such as pathogens transmission and host‐pathogen co‐evolution, as illustrated by transmissible cancer in *Mytilus* spp., which spread from the Northern to the Southern hemisphere most likely through biofouling on boats (Yonemitsu et al., 2019).

BOX 3 **“Biological portuarization”: Life‐sized evolutionary experiments for the marine environment**

We here define “biological portuarization” as the evolution of marine species in port‐ecosystems under human‐altered selective pressures, by analogy with the urbanization process on land that creates novel selective pressures for resident species (Szulkin et al., 2020), with domestication in agro‐ecosystems (Larson & Fuller, 2014), or with pestification (Saleh et al., 2014), the co‐evolution of pathogens in relation to the selection of crop or animal lineages during domestication. Portuarization is a word that does not yet exist in English but does exist in French (“portuarization”) and means “to give a port characteristic to”. We believe it is useful to introduce new terminology to describe each type of human‐induced evolution in order to better delineate it, study it, and communicate about it, if, as we suspect, it proves to be a threat to biodiversity. The study of biological portuarization is a stimulating way to investigate fast evolutionary responses to human‐altered environments, in particular facing increasing coastal hardening associated with boating and shipping (Floerl et al., 2021).

Experimental evolution provides a precious lens on evolutionary processes, but this only applies to short life‐cycle organisms, in a simplistic environment, and during very short adaptive pulses (Kawecki et al., 2012). Alternatively, the study of domestication is also a fruitful area of investigation, providing evolutionary experiments that unfold over longer periods (Larson & Fuller, 2014). For similar reasons, urban evolution has recently become a fertile area of research, providing replicates of adaptation to urbanized environments (Santangelo et al., 2022; Szulkin et al., 2020). Fisheries‐induced evolution, which typically results in smaller fishes with a younger age at maturity due to size‐selected catches by fishing gears (Ernande et al., 2004), is also a nice example of human‐induced evolution providing useful information and allowing to calibrate evolutionary models. However marine evolutionary sciences have to date little followed this idea of identifying and investigating life‐size experiments derived from human activities. Ports provide such life‐sized experiments to better examine ongoing evolutionary processes, and thus better calibrate theoretical models and fine‐tune our projections. We argue that ports are (understudied) Darwinian arenas that provide replicates of life‐sized evolutionary experiments.

Ports can be compared with “giant mesocosms” where species develop, survive, and reproduce in novel and singular environments, within uniquely new species assemblages with invasive and native taxa coexisting. Humans have built ports on all the coasts of the world, allowing people to travel and to develop trade. Since the second half of the 20th century, maritime traffic has strongly intensified and ports have become larger and more numerous. These are trends that are not expected to fade (Bugnot et al., 2021; Jouffray et al., 2020; Sardain et al., 2019), while also providing replicated evolutionary playgrounds across the world (Santangelo et al., 2020). We are advocating for the development of research dedicated to examining biological portuarization.

In addition to the intrinsic value of studying evolutionary processes in ports for its fundamental understanding, we also highlighted potential applications for nonindigenous species management and conservation, and putative threat that port populations could impose on wild populations. For instance, we pointed out that enhanced controls of the opening between the port and seaside waters could promote gene swamping in portuarized populations, and that the coupling between local adaptation clines and intrinsic isolation clines could slow down the spread of invasive species. Altogether, we argue that evolutionary processes in ports can play a major role regarding conservation issues and should be better taken into account in management decisions.

There are considerable opportunities to fill several knowledge gaps. First, to date, only a few evolutionary studies have been dedicated to marine urban environments in general (Alter et al., 2021), and ports in particular. Indeed, marine population genetics has long been mainly interested in natural populations, and sampling is generally carried out outside urbanized environments. Consequently, there is an urgent need for increased screening of ports in population genetic studies to uncover shifts in connectivity (Section 2), mechanisms sustaining local adaptations (Section 3), and/or cases of anthropogenic hybridization (Section 4). Second, ports are most often considered as homogenous entities, while they are more likely to be equally environmentally heterogeneous as the urban mosaic itself. This has been readily documented by ecological studies that showed specific communities associated with particular port microhabitats (e.g., floating pontoons vs. pilings, Leclerc et al., 2020), and should therefore be taken into account in the context of evolutionary hypothesis testing. Experimental set‐ups will have to be carefully designed to account for port properties. Third, experimental approaches to describe particular port phenotypes are currently lacking. This is fertile ground for future research. Demonstrating the availability of distinct port phenotypes (or phenotypes pertaining to distinct microhabitats of the port environment, see Cheptou & Lambrecht, 2020, for a terrestrial example) may indeed be key to test adaptive or acclimation processes, and to better understand the outcomes of anthropogenic hybridization. Fourth, the putative threats due to escapees of portuarized genotypes in natural populations also deserve dedicated studies, including joint investigation of adjacent natural and port habitats, both contributing to an ever‐changing coastal network.

To conclude, the spatially repeated port singularities make these marine urban habitats perfect arenas for evolutionary studies, including large‐scale in situ experiments, which can be compared with “giant mesocosms”. The use of ports as field labs for evolutionary studies will be strongly dependent on (i) knowledge of the environmental conditions within and between ports, (ii) the ability to compare these conditions with those pertaining to the native ranges of the species under study, and (iii) the need to properly quantify propagule pressure. Large‐scale and time‐detailed ecological conditions are accessible through remote sensing (temperature, salinity, chlorophyll, etc.; Lecours et al., 2021), but their availability at the scale of ports appears to be limited. Additionally, propagule pressure is a difficult parameter to estimate and relies on surveys, proxies, and model estimations (e.g., Drake et al., 2015). Therefore, interdisciplinary research that integrates port ecology, transport networks, and evolution will be necessary to tackle the questions we have developed in this paper.

## CONFLICT OF INTEREST

None to declare.

## Data Availability

Data sharing is not applicable to this article as no new data were created or analyzed in this study.
